# Emerging pathogen evolution: Using evolutionary theory to understand the fate of novel infectious pathogens

**DOI:** 10.15252/embr.202051374

**Published:** 2020-08-30

**Authors:** Camille Bonneaud, Ben Longdon

**Affiliations:** ^1^ Centre for Ecology and Conservation, Biosciences University of Exeter Penryn Cornwall UK

**Keywords:** Ecology, Microbiology, Virology & Host Pathogen Interaction

## Abstract

Evolutionary biology is key to potentially predicting virulence and transmission after a pathogen jumps into a new host species. This knowledge would be valuable for designing public health strategies.

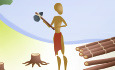

What needs to happen for a pathogen to successfully infect a new host species? This is a critical question given the devastation that emerging pathogens can cause. For instance, several major pandemics have occurred in the last century that have killed millions of people, including the pandemics of 1918 H1N1 influenza, which partly originated in birds, and of HIV, which originated in chimpanzees. The 21^st^ century has already had its share of outbreaks of zoonotic origin—SARS, MERS, Ebola, Hendra and Nipah—and the current COVID‐19 pandemic has become a major international public health, social, economic and political crisis. Steady advances in biology and medicine mean that we are now better at identifying pathogens of epidemic potential and at monitoring the spread of novel infections worldwide than ever before (e.g. https://nextstrain.org/). Nevertheless, designing effective public health strategies to contain infectious outbreaks, particularly given other potentially conflicting interests (e.g. economic), remains incredibly complex. Here, we provide a brief overview of our current understanding of how and why pathogens evolve in novel host species, to guide an understanding of the potential evolutionary consequences of our control measures.

## Jumping into a novel host

A successful jump into a novel host species is a three‐step process. First, the pathogen must come into contact with the novel host. Next, it must successfully infect the novel host, which may involve binding to host cell receptors, entering cells and hijacking the cellular machinery to replicate, and/or escaping host defences. Finally, there must be sufficient onwards transmission of the pathogen for its persistence and spread through the novel host species.

Steady advances in biology and medicine mean that we are now better at identifying pathogens of epidemic potential and at monitoring the spread of novel infections…

The steps of infection and transmission can represent such formidable challenges for the pathogen that, in most cases, it fails to establish in the novel host species. A pathogen may rely on the host's cellular machinery for survival and replication, and to persist, it needs to avoid the host's immune defences. Even in an environment to which the pathogen is well adapted, this can be a feat, but it is likely to be even more so in a novel host where the cellular machinery and defences are unknown. The relative similarity of these conditions in closely related species may explain why pathogens are more likely to successfully shift to a new host species closely related to original donor host. For example, more zoonotic human pathogens have originated from non‐human mammals than from other vertebrate taxa and several major human viruses, such as hepatitis B virus, HIV and yellow fever virus, were originally acquired from primates (Parrish *et al*, 2008). The novel host may also present unexpected difficulties for transmission if differences in behaviour and/or the social structure affect contact rates between individuals. To overcome all these challenges, the emerging pathogen will need to acquire critical mutations that maximise its success in a novel host species.

**Figure 1:**
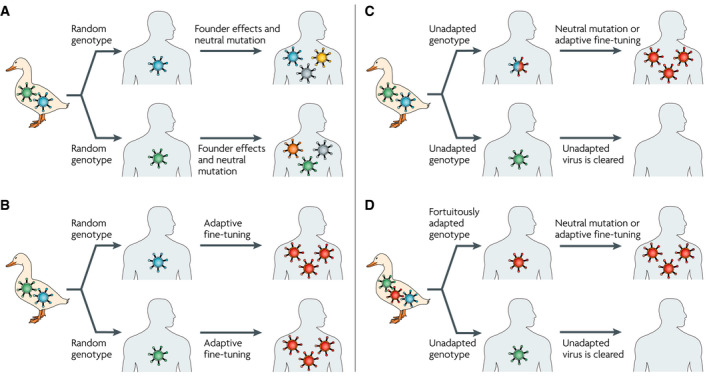
Different mechanisms of pathogen emergence in novel host species (A) The pathogen has “off the shelf” or pre‐existing adaptations that have arisen in the original host species and allow direct infection and transmission within the novel host. The distribution of pathogen variants in circulation in the novel host species will then depend on which variant(s) successfully jumped into the new host and on the neutral mutations that have accumulated subsequently. (B) Although the pathogen harbours the necessary pre‐existing adaptations for initial infection and transmission within the novel host species, longer‐term persistence requires the acquisition of mutation(s) that fine‐tune the processes of infection and/or transmission in the novel host environment. Thus, the pathogen variants found in the novel host species are those that exhibit such adaptive fine‐tuning. (C) Following transmission between original and novel host species, the pathogen is initially unable to persist within the novel host. Persistence will depend on the acquisition of adaptive mutations that allow infection and transmission within the novel host. (D) Only one or a few variants of the pathogen have pre‐existing adaptations to the novel host species. As a result, inter‐species transmission events only give rise to pathogen emergence in some cases, with pathogen variants in the novel host subsequently shaped by both adaptive fine‐tuning and neutral mutations. Adapted from Pepin *et al* (2010) with permission.

While some emerging pathogens may have “off‐the‐shelf” or pre‐existing adaptations that allow them to infect and transmit in a different host species, others rely on the ability to rapidly evolve such adaptations after jumping into a novel host (Pepin *et al*, 2010) (Fig 1). The extent to which the critical mutations necessary for sustained transmission in a novel host need to have pre‐existed or can arise *de novo* remains, however, unknown. Regardless, following a jump into a novel species that then becomes the predominant host—in contrast to occasional spillovers—a pathogen will typically undergo evolutionary changes that, if anything, should fine‐tune its adaptation to the novel environment.

The steps of infection and transmission can represent such formidable challenges for the pathogen that, in most cases, it fails to establish in the novel host species.

Such adaptive changes occur through mutations that improve the process of infection or increase transmission. These can include mutations that strengthen the binding affinity to the novel host cells or tissues, enable the pathogen to avoid, manipulate or suppress the host immune system, and/or give rise to more effective transmission, such as by increasing persistence in the external environment. For example, influenza viruses naturally infect birds through a faecal–oral route. Yet a change in their sialic acid‐binding preference can allow them to bind and enter human epithelial cells of the upper respiratory track. Gain‐of‐function experiments with avian H5N1 influenza A, which infects but does not efficiently transmit among humans, showed that only a handful of mutations would be sufficient to increase transmissibility via respiratory droplets in a mammalian host. The number and position of the mutations required to improve infection and transmission in the novel host will affect the likelihood and speed of pathogen adaptation following a host shift, with critical implications for pathogen persistence and virulence.

## Virulence at disease outbreak

Virulence is stringently defined as the disease‐induced mortality rate of infected hosts. More virulent infections cause greater harm and/or death. There is obviously a large variation in the amount of harm that different pathogens will cause, ranging from typically benign (common cold) to highly lethal (Ebola). But there is also variation in the amount of harm caused by different variants of the same pathogen. Furthermore, the host's response also shapes the overall level of virulence of an infection. A healthy person with a working immune system should suffer less from an infection than someone with compromised immunity, such as HIV‐infected patients or those undergoing chemotherapy for cancer treatment. Moreover, earlier population exposure and acquired immunity also determine the extent to which the immune system is able to fight an infection. European settlers brought to the Americas terrible diseases, most notably smallpox, that ravaged native populations but that they themselves were largely immune to either because of previous exposure, or because they had inherited natural resistance evolved over time. Since virulence is an outcome of the interaction between pathogen and host, the level of virulence of an infection may therefore differ across circulating pathogen variants, host species, populations and even individuals.

The virulence of a novel infection immediately after a host shift is therefore difficult to predict. Classically, it was thought that the initial maladaptation of host and pathogen to each other resulted in highly virulent infections, evolving towards mutualisms in the longer term. While the latter has long since been shown not to be the case, the belief that most novel infections are initially more virulent is largely due to an ascertainment bias towards the most virulent outbreaks, while infections causing little or no damage often go undetected.

Since virulence is an outcome of the interaction between pathogen and host, the level of virulence of an infection may therefore differ across circulating pathogen variants, host species, populations and even individuals.

Determining the level of virulence necessary for novel pathogen emergence is challenging and requires comparison between donor and recipient host species, a task often limited by a lack of knowledge of the donor species. Nonetheless, a comparative study of human viruses suggested that infections with lower virulence were more likely to establish sustained transmission among humans than more virulent ones (Geoghegan & Holmes, 2018). In accordance, the high virulence of the Nipah and Ebola viruses—alongside strict public health interventions—may partly explain why their outbreaks exhibited only short stuttering chains of transmission before ultimately dying out of their own accord. Further work is required to determine whether pathogens that establish infections of lower virulence in novel hosts are, indeed, more likely to successfully adapt than more virulent ones.

Whatever the level of virulence following a host shift, we should expect to see differences across host species, populations and individuals, which are caused by a combination of environmental and genetic effects. That related host species tend to show similar levels of virulence can help predict the level of virulence in closely related species. Such an approach has been employed, for example, to identify the amphibian species particularly at risk of extinction from the devastating chytrid fungus, *Batrachochytrium salamandrivorans*.

Within a given host species, differences in the ability to resist novel infections may stem from disparities in access to food and, for humans and livestock, in access to health care, or from previous exposure to the pathogen. For instance, during the 1918 H1N1 influenza pandemic, some US cities were spared during the first wave of infection. Those cities, however, suffered higher mortality rates during the second, more severe wave of infection, presumably because people in previously affected cities had acquired immunity a few months earlier. Past pathogen exposures may also shape the host population's ability to resist novel infections. The natural resistance of a small proportion of Europeans to HIV has been hypothesised to be a hand‐down from the Black Death in the Medieval Ages: selectively beneficial mutations at that time would now provide some level of cross‐immunity to HIV. While this is unlikely to be the case in this particular instance, such cross‐protection may, nevertheless, explain why elderly people who would have been exposed to pre‐1950 influenza strains were less affected than other age groups by the 2009 swine H1N1 influenza outbreak. Either way, any variation in host responses to novel pathogens, combined with variation in the level of virulence of the pathogen at outbreak, is expected to have repercussions for subsequent pathogen evolution.

## Modelling virulence evolution

**Figure 2:**
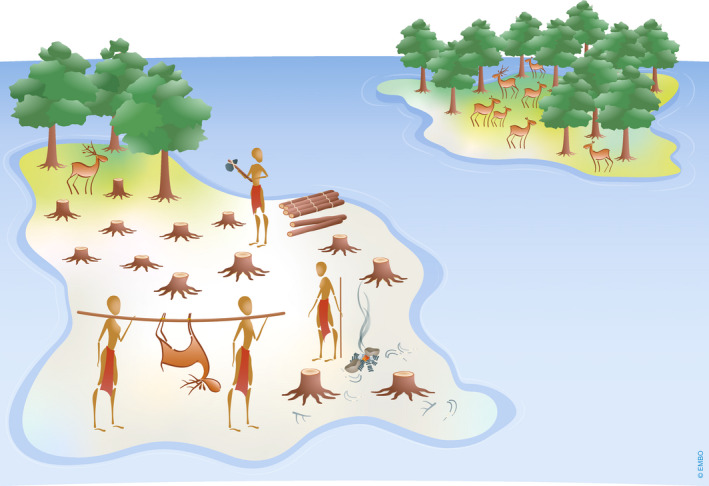
Illustration of a trade‐off between exploitation and movement In order to replicate and transmit, pathogens need to exploit their host but in doing so, they can kill them and so reduce the time window to transmit to another host individual. The figure shows an analogous problem faced by early island communities when environmental exploitation was required for dispersal to new islands, but simultaneously reduced the time available to do so.

During the past decades, it has become clear that evolutionary theory can provide a powerful framework for understanding why emerging pathogens harm their hosts and how virulence may change over time (Anderson & May, 1982). Infection‐induced symptoms and host mortality do not arise because pathogens “want” to harm their hosts; rather, harm occurs through two non‐antagonistic processes. First, pathogens exploit their hosts to persist and replicate; in a similar way as we degrade our environment when we use its natural resources to subsist and reproduce. Second, symptoms are sometimes necessary for pathogen transmission; sneezing and coughing allow the transmission of common cold and flu viruses to another host. To use the analogy again, this is akin to humans cutting down a forest for making ships to disperse to new islands (Fig 2). The harm caused to the host, however, means that a pathogen must balance the advantage of host exploitation for replication and/or transmission, with the cost of killing the host too quickly, and so reduce the window of opportunity for transmission to another host (Alizon *et al*, 2009). To continue with the analogy, this would be equivalent to humans exploiting their environment while having to balance its rate of degradation with opportunities for moving to other suitable habitats.

… evolutionary theory can provide a powerful framework for understanding why emerging pathogens harm their hosts and how virulence may change over time.

With this in mind, evolutionary theory can be used to model optimal pathogen virulence under a given set of conditions. Although we have seen that a host population is unlikely to be universally susceptible to a novel pathogen, let us assume for simplicity that most individuals are susceptible and have high contact rates. Under this crude scenario, pathogen variants with higher virulence should have a selective advantage. It should not matter how fast the pathogen kills its host, because the large number of susceptible hosts means that there is plenty of opportunities for transmission, even if the infection is short. Another reason is that, if the prevalence of infection is high, it becomes more likely that different pathogen variants emerge, which compete with each other during co‐infection. Since natural selection should favour the variant that is able to transmit faster, thereby outcompeting the others by killing the host before others can transmit, virulence should, under those conditions, increase. Whether the virulence of an emerging pathogen actually peaks following a host shift will depend on the starting level of virulence, the cost of high virulence and any barriers to transmission.

## Barriers to transmission

A newly emerged pathogen can hit barriers to transmission if there are less host individuals to infect. This can happen when susceptible individuals become rare, either because most have died from infection leaving mainly naturally resistant individuals, and/or because those that have survived and recovered have acquired immunity that prevents reinfection. Barriers to transmission can also arise through behaviour to minimise contamination, such as avoiding sick individuals. For example, dead or diseased larvae and pupae of the honeybee *Apis mellifera* are removed to protect the nest from infection. Under such conditions of reduced transmission opportunities, the balance should tip in favour of less virulence to increase the window for what has now become rarer transmission opportunities. In terms of virulence optimum, this means that the most evolutionarily successful pathogens should do less harm, and thereby have more time to transmit. In support, a model of an H5N1 pandemic found that public health strategies of physical distancing could exert strong selection in favour of viral variants of lower virulence that survived longer within their hosts. Similarly, models of the evolutionary impacts of different types of vaccines have shown that vaccines that prevent infection or block pathogen transmission will select for lower virulence (Gandon *et al*, 2001). However, while blocking infection and transmission should lead to a reduction in virulence over time, strategies that purge the pathogen after it has established an infection are predicted to lead to the opposite evolutionary outcome.

Clearing the pathogen should indeed select for increased virulence because clearance reduces the duration of infection, hence tipping the balance in favour of faster transmission before the pathogen is eliminated. The jump of the bacterial pathogen *Mycoplasma gallisepticum* from its original poultry host into a wild North American songbird in 1994 caused an epidemic so intense that naturally resistant individuals quickly increased in frequency over the subsequent years (through increased survival and/or reproduction). Such rapid spread of host resistance, mediated by immune clearance, was, in turn, found to have driven the rapid evolution of higher pathogen virulence and transmission rates (Tardy *et al*, 2019).

… while blocking infection and transmission should lead to a reduction in virulence over time, strategies that purge the pathogen […] are predicted to lead to the opposite evolutionary outcome.

While pathogen evolution in response to host immunity can be challenging to detect in wild populations, similar patterns of evolutionary change can be observed, for example, in response to our use of antibiotics. In a mouse model, infections with the diarrhoeal bacterial pathogen *Salmonella typhimurium* can contain a mix of virulent inflammatory‐causing pathogen variants and avirulent cheats that avoid the cost of triggering inflammation. Antibiotic treatment was found to favour the virulent variants over avirulent ones, thus leading to increasing virulence and transmissibility over time (Diard *et al*, 2014). The consequences of increasing pathogen virulence may not be problematic for hosts that can deal with it through immunity or medication. Complications, however, arise when individuals differ in their abilities to defend themselves: the evolution of more virulent pathogens would be potentially catastrophic for hosts that are less resistant.

## How easy it is for a pathogen to adapt to a novel host?

Evolutionary theory can therefore be used to model the fate of emerging pathogens under different conditions. But how easy is it for an emerging pathogen to evolve adaptively and how fast can it do so? The answer is that it will depend on the likelihood and speed of acquiring the necessary mutations (Woolhouse *et al*, 2005). References to “mutating pathogens” in the science fiction literature and tabloid media usually trigger panic. However, mutation is simply a property of the error‐prone replication of pathogens, as mismatches are introduced during the duplication of the genome. Because most mutations will adversely affect the pathogen and be quickly removed by natural selection, pathogens replicating with a high mutation rate, a small genome size—where mutation will have large functional effects—and a large population, should have the highest adaptive potential. In support, RNA viruses, which have all three of those traits, are more likely to host shift than other types of pathogens. Bacteria, which typically have lower mutation rates, large genome sizes and lower census populations, may be more limited in their ability to adapt to novel hosts. It is worth noting that the few known examples of bacterial host shifts involve pathogens with unusually small genome sizes and high mutation rates such as *Staphylococcus aureus* or *M. gallisepticum*. Either way, the likelihood that functionally beneficial mutations arise will be greater the more host individuals are infected.

When evolutionary concepts are integrated into such intervention strategies, however, they can lead to dramatic progress in the fight against diseases.

But even when beneficial mutations do arise, they will not necessarily lead to evolutionary change. One reason is that transmission between host individuals can create bottlenecks with only a few pathogen variants able to establish an infection. If these bottlenecks are stochastic and randomly remove some of the genetic diversity that has arisen during an infection, then any new beneficial mutation may be lost by chance through genetic drift. For example, new HIV infections are typically established by one or very few viruses, even though the donors may harbour a highly diverse viral population. In addition, there may be host population‐level processes that accentuate stochasticity around the transmission of any new mutation if, for example, there are spatial heterogeneities or seasonal variation in host densities.

Even if the beneficial mutation successfully transmits, the small size of the founding pathogen population at the onset of infection means that the growth of the different variants will also be subject to chance. Any beneficial mutation risks, then again, to be lost through genetic drift. Finally, a new beneficial mutation can also be eliminated by the host immune system, either because it is cleared directly or because, by reducing the pathogen population size, immune clearance increases once more the chances that the mutation will be lost through drift. There are other effects that can slow down or even hinder the spread of a beneficial mutation, and while these effects may sometimes be overcome by the selective gains conferred by that mutation, it is clearly not so simple for a pathogen to evolve adaptively to its novel host.

## Conclusion

Although an evolutionary framework enables us to make key projections regarding the fate of novel infectious pathogens, including in response to our control measures, evolutionary biology is still too often overlooked in medical and public health interventions (Nesse & Stearns, 2008). When evolutionary concepts are integrated into such intervention strategies, however, they can lead to dramatic progress in the fight against diseases. One of the best illustrations of the power of evolutionary theory is our response to antibiotic resistance in bacterial pathogens. As clinical antibiotics are derived from microbial toxins that bacteria use to fight each other, the evolution of resistance is therefore simply a natural phenomenon that we have unfortunately nurtured through extensive use of antibiotics in health and agriculture. The modelling and experimental testing of evolutionary outcomes not only revealed that methods of antibiotic administration intuitively believed to minimise such evolution actually worsen it, but also helped to design more effective alternatives of antibiotic usage. A second example is the fight against malaria, for which our ability to generate control measures that will not erode over time—that are “evolution proof”—is improved by an understanding of the evolutionary ecology of its mosquito‐vector. Such measures may range from using microsporidian parasites to decrease larvae survival, adult biting rate and longevity, to insecticides targeting old and/or malaria‐infected mosquitoes for which selection on resistance is likely to be weak. The recent incorporation of evolutionary biology in cancer research has also uncovered its value for improving health beyond infectious diseases. By providing a deeper understanding of the behaviour and responses of infectious pathogens, evolutionary biology thus represents a powerful approach for tackling human, animal, plant and even ecosystem health.
